# Vaccines in Congenital Toxoplasmosis: Advances and Perspectives

**DOI:** 10.3389/fimmu.2020.621997

**Published:** 2021-02-15

**Authors:** Mariana Barros, Daniela Teixeira, Manuel Vilanova, Alexandra Correia, Natercia Teixeira, Margarida Borges

**Affiliations:** ^1^Departamento de Ciências Biológicas, Faculdade de Farmácia, Universidade do Porto, Porto, Portugal; ^2^Immunobiology Group, Instituto de Investigação e Inovação em Saúde (i3S), Universidade do Porto, Porto, Portugal; ^3^Instituto de Biologia Molecular e Celular (IBMC), Universidade do Porto, Porto, Portugal; ^4^Departamento de Imuno-Fisiologia e Farmacologia, Instituto de Ciências Biomédicas Abel Salazar (ICBAS), Universidade do Porto, Porto, Portugal; ^5^Applied Molecular Biosciences Unit/Rede de Química e Tecnologia (UCIBIO/REQUIMTE), Departamento de Ciências Biológicas, Faculdade de Farmácia, Universidade do Porto, Porto, Portugal

**Keywords:** pregnancy, toxoplasmosis, congenital, vaccination, maternal-fetal

## Abstract

Congenital toxoplasmosis has a high impact on human disease worldwide, inducing serious consequences from fetus to adulthood. Despite this, there are currently no human vaccines available to prevent this infection. Most vaccination studies against *Toxoplasma gondii *infection used animal models in which the infection was established by exogenous inoculation. Here, we review recent research on potential *T. gondii* vaccines using animal models in which infection was congenitally established. Endeavors in this field have so far revealed that live or subunit vaccines previously found to confer protection against extrinsically established infections can also protect, at least partially, from vertically transmitted infection. Nevertheless, there is no consensus on the more adequate immune response to protect the host and the fetus in congenital infection. Most of the vaccination studies rely on the assessment of maternal systemic immune responses, quantification of parasitic loads in the fetuses, and survival indexes and/or brain parasitic burden in the neonates. More research must be carried out not only to explore new vaccines but also to further study the nature of the elicited immune protection at the maternal-fetal interface. Particularly, the cellular and molecular effector mechanisms at the maternal-fetal interface induced by immunization remain poorly characterized. Deeper knowledge on the immune response at this specific location will certainly help to refine the vaccine-induced immunity and, consequently, to provide the most effective and safest protection against *T. gondii *vertical infection.

## Introduction

*Toxoplasma gondii* is an obligate intracellular protozoan parasite and the etiologic agent of congenital toxoplasmosis. *T. gondii* is considered one of the most successful parasites worldwide, infecting over 30% of the human population, with high associated disease burden (1, 2). Seroprevalence varies greatly from region to region, ranging from approximately 30% in the American, European, and Asiatic regions, to more than 60% in the African continent (3, 4). The disease is potentially dangerous in women who become infected during pregnancy, as it can lead to transplacental transmission of the parasite upon primary infection or re-infection with highly virulent strains (5, 6). The incidence of congenital toxoplasmosis varies according to the timing of infection during pregnancy. The transmission rate is greater in the final stages of pregnancy, but the severity of infection is greater in early gestation (7). In the more severe cases, hydrocephalus, chorioretinitis, and cerebral calcification may occur, according to the parasite’s brain and ocular tropism (5). An association between congenital infection and the development of neurological and psychiatric disorders later in life, including schizophrenia, Alzheimer’s disease, bipolar disease, and even suicidal tendencies has also been suggested (8, 9).

Innate and adaptive immunity determines protection against *T. gondii* infection. An effective immune response must control parasite growth while avoiding immunopathology. In both mice and humans, the IL-12- IFN-γ axis is the main immune mechanism responsible for parasite control. Protection mediated by IFN-γ produced by NK and Th1 cells induces the expression of immunity-related GTPase and/or guanylate-binding proteins, indoleamine-2,3-dioxygenase, and NO production. TNF-α has also been associated with host protection, as highlighted in patients with defective IFN-γ signaling (10). TGF-β, IL-6, and IL-23 promote the production of IL-17 that may also play a host protective role in toxoplasmosis by avoiding excessive IFN-γ-dependent inflammation (11). T cells producing IL-10, which include T regulatory cells and Foxp3^-^T-bet^+^ Th1 cells, can limit excessive inflammation driven by *T. gondii* (12). A regulatory role for IL-4, and IL-27 in minimizing host tissue injury due to exacerbated inflammation has also been shown (10, 13). A good vaccine candidate would thus induce not only IL-12 and IFN-γ, but also counterbalancing cytokines such as IL-4, IL-10, and IL-27 (14).

It is important to note that there are no licensed human vaccines able to prevent toxoplasmosis (15). The lack of effective treatment makes the development of a vaccine against congenital toxoplasmosis one of the main objectives in the management of this disease. Here, recent findings in vaccination approaches to congenital toxoplasmosis using animal models of vertical *T. gondii* infection will be reviewed and the possible implications in the quest for a vaccine protecting from congenital toxoplasmosis will be discussed.

## *In Vivo* Models to Study Congenital Infection

In the study of toxoplasmosis, animal models are used to better understand the disease pathology and the immunological mechanisms induced by infection, as well as to assess the effectiveness of experimental vaccination. However, no single animal model has, so far, been able to mimic all clinical symptoms and signs developed by humans in response to *T. gondii* infection (16). For congenital toxoplasmosis, the murine model is commonly used as it allows a short pregnancy period and mimics features of human congenital toxoplasmosis, namely the co-localization of inflammatory cells and necrosis at the maternal-fetal interface after primo-infection during pregnancy (17). Further, primo-infection confers resistance to maternal fetal transmission throughout later infections (16). However, the immune response to *T. gondii* in mice and humans presents distinct features and this should be adequately considered (18, 19).

Most vaccination studies in congenital infection reviewed here used Kunming, BALB/c, Swiss OF1, and CBA/J mice. No study was found using the C57BL/6 mouse strain. This might be due to excessive susceptibility to disease exhibited by *T. gondii* infected C57BL/6 mice. In contrast, BALB/c mice present higher resistance to infection, more closely resembling humans, rendering this strain more suitable for vaccination studies in congenital infection models (20–22). In murine congenital toxoplasmosis, C57BL/6 mice exhibited higher abortion rate compared to BALB/c due to exacerbated proinflammatory cytokines such as TNF-α (23). Mouse strain differences in major histocompatibility complex haplotypes (*e.g.*, H-2^b^, H-2^d^, H-2^k^ for C57BL/6, BALB/c, and CBA/J, respectively), and therefore, antigenic presentation, could explain different susceptibility to *T. gondii* infection and induced immunopathology (20).

Other animal models can be used such as sheep, rats, guinea pig, or hamster (24–28). Rats and sheep are widely used in studies addressing drug effectiveness to *T. gondii* (18). Rats have placental development and hemochorial placentation identical to humans (29). In sheep, congenital toxoplasmosis is very similar to what occurs in humans (15). Thus, sheep is an adequate animal model to study congenital toxoplasmosis, not only because it shares important aspects with the human infection, but also because it directly contributes to the study of new disease control measures in livestock, also severely affected by *T. gondii* (15). There are well established models of toxoplasmosis in pregnant sheep that provide a starting point for the preparation and testing of new vaccines (15).

## Vaccines in Vertical Infection

This section aims to describe the vaccines already tested in congenital infection models, and to reveal the gap of analysis concerning immune cells and related mechanisms induced by immunization at the level of maternal-fetal interface as wells as in the neonates. Literature research was performed through a PubMed search, using the query “[(*Toxoplasma gondii*) AND (vaccine)] AND (congenital toxoplasmosis [title/abstract])”. The results presented pertain to studies using pregnant mice where protection and immune responses of pregnant mice and their offspring was evaluated.

### Live-Attenuated Vaccines

Live-attenuated vaccines have been the most studied in the context of congenital transmission (**Table 1**). These vaccines consist of parasites with reduced virulence but are nevertheless capable of inducing an immune response (36). Alternatively, attenuated virulent strains can also be used, albeit, in this case, the attenuation must be complete to ensure that the vaccine will not cause the disease (37). These vaccines present several advantages, such as using whole parasites, meaning that multiple antigens are available simultaneously. Live vaccines also do not usually require repeated immunizations or the use of adjuvants. However, a concern in using this type of vaccination is the possibility of reversing the parasite to a virulent state causing infection (36). Moreover, live vaccines are not recommended to be used in immunocompromised hosts. Toxovax®, the only licensed vaccine for toxoplasmosis, administered to avoid abortion in sheep, is a live-attenuated vaccine, using the strain S48 tachyzoites, originally isolated from an aborted lamb in New Zealand (14). This vaccine is not licensed for humans due to the possibility of parasite reversion to its virulent form (38). Moreover, it has a short shelf life and does not lead to full parasite elimination (14).

**Table 1:** Live-attenuated vaccines tested in vertical *Toxoplasma gondii* infection models.

Strain/animal	Immunogen/strain/delivery	Day of mating post-immunization	ChallengePregnancy day/route/#parasite form/Strain	Dam sacrifice	Maternal parameters	Progeny parameters	Highlighted results	References
Swiss OF1/mice*	20 tachyzoites Δ*mic1-3*/ RH/ ip	Day 60	Day 11/oral/ 45 cysts/ 76K strain	-Day 17 of pregnancy (exp#1) -1 month after delivery (exp#2)	-Toxoplasma specific IgG levels *-Ex-vivo* splenocyte specific cytokine production (exp#1) -Brain cyst load (exp#2)	-Fetus parasite load (exp#1) -Survival of neonates and mean weight of pups at day 11 after birth -Brain cyst load at day 35 after birth (exp#2).	-Potent maternal humoral and Th1 responses. -Decreased fetus parasite load and, maternal and neonate brain parasite load. -100% Neonate survival and higher pups weight.	(30)
Bizet, Romanov, and Solognot/ewes**	10^5^(exp#1), 2x10^6^(exp#2), and 10^5^ (exp#3) tachyzoites Δ*mic1-3*/ RH/ sc (exp#1, #2) and ip (exp#3)	Day 60	~ Day 75 (mid gestation)/oral/ 400 sporulated oocysts (exp#1, #2), 100 sporulated oocysts (exp#3)/ PRU strain	~ Day 135 of pregnancy (exp#1, #3)	-Seroconversion after immunization and before pregnancy (exp#1, #2, #3) -Evaluation of infection clinical signs (febrile response) (exp#1, #2, #3) -Brain cyst load (exp#1, 3)	-Number of non-viable and viable lambs. -Evaluation of abortion. -Mean weights of viable lambs. -Brain cyst load in lambs.	-Maternal seroconversion and lower febrile response. -Protection against *T. gondii* abortion and higher rate of viable lambs. -Decreased brain parasite load in vaccinated ewes and in the lambs.	(31)
Kunming/mice	5x10^4^ tachyzoites Δ*gra17*/ RH/ ip	Day 70	-Day 12/oral/ 10 Cysts/Pru strain (exp#1) -Day 18/ip/ 200 tachyzoites/ RH strain (exp#2)	-Day 18 of pregnancy (exp#1) -Day 30 after delivery (exp#2)	*-Ex-vivo* splenocyte specific cytokine production (exp#1) -Brain cyst load (exp#2)	-Litter size and survival of delivered pups at birth and 5 days after birth (exp#2). -Body weight 35 days after birth (exp#1) and 5 days after birth (exp#2). -Brain cyst burden in pups at 35 days after birth.	-Higher maternal IFN-γ, IL-12, and IL-10 productions. -Decreased maternal brain parasite load and no clinical signs in immunized dams. -Protection against *T. gondii* abortion. -Higher litter sizes of viable neonates, survival rate and body weight. -Decreased pup brain parasite load.	(32)
Kunming/mice	10^6^ tachyzoites Δ*gra17*Δ*npt1*/ RH/ ip	Day 60	Day 5/oral/ 10 cysts/ Pru strain	Day 30 after delivery	Brain cyst load.	-Litter size and survival rates of delivered pups at birth and 30 days after birth - Body weight 30 days after birth. -Brain cysts burden in pups at 30 after birth.	-Decreased maternal brain parasite load. -No abortions in immunized mice. -Increased litter size, pups survival rate, and body weight.	(33)
Kunming/mice	500 tachyzoites Δ*cdpk*2/ Pru/ ip	Day 70	Day 12/oral/ 10 cysts /Pru strain	-Day 18 of pregnancy (exp#1) -Day 5 after delivery (exp#2)	*-Ex-vivo* splenocyte specific cytokine (exp#1). -Brain cyst load (exp#2).	-Litter size and survival of delivered pups at birth and 35 after birth. - Body weight 35 days after birth. - Brain cysts burden in pups at 35 after birth.	-Higher maternal IFN-γ, IL-2, IL-12, and IL-10 production. - Decreased maternal brain parasite load. - Higher litter sizes of viable neonates, survival rate, and body weight. -Decreased pup brain parasite load.	(34)
Kunming/mice	10^6^ tachyzoites Δ*tkl1*/ RH/ ip	Day 60	Day 5/oral/ 10 oocysts/ Pru strain	Day 30 after delivery	Brain cyst load	Evaluation of abortion.	-Healthy dams and decreased maternal brain parasite load. - Protection against *T. gondii* abortion. - Higher litter sizes of viable neonates.	(35)

*Mice: pregnancy length of 21 days; ** ewes: pregnancy length of 152 days. MIC1-3KO: micronemal protein 1 and 3 double deletion mutant; ΔGRA17: dense granule protein 17 deletion mutant; Δgra17Δnpt1/: dense granule protein 17 (gra17) and novel putative transporter (npt1) double deletion mutant; ΔCDPK2: calcium-dependent protein kinase 2 (cdpk2) deletion mutant; Δtkl1: tyrosine kinase-like 1 (tkl1) deletion mutant. RH strain (type I); 76K and PRU strains: type II: ip: intraperitoneal; sc: subcutaneous. exp#1: experimental design 1; exp#2: experimental design 2.

Live-attenuated vaccines may be produced by gene targeted approaches. Such is the case of a modified RH strain lacking two genes, respectively encoding micronemal protein 1 (MIC1), which associates to MICs 4 and 6, rendering them active, and MIC3, a micronemal protein necessary for host cell invasion and MIC8 function. The double deletion of these genes (Δ*mic1-3*) resulted in the loss of function of these five proteins. Female Swiss OF1 mice immunized with Δ*mic1-3* strain exhibited higher levels of IFN-γ and IL-2 and a smaller number of brain cysts compared to non-immunized mice when infected with *T. gondii*. Also, all pups born from immunized animals survived compared to 64% of non-immunized mice. Moreover, 55% of the pups born from immunized mice did not present brain cysts and those with brain cysts exhibited a 91% reduction of cyst burden (30). This vaccine was also tested in Bizet, Romanov, and Solognot ewes, using the same experimental design but adapted to the ewes’ pregnancy length. It showed to be effective by both subcutaneous (sc) and intraperitoneal (ip) routes, inducing protection against abortion, a higher rate of viable lambs and a decrease of brain parasite cysts in the lambs born from vaccinated ewes (31). Wang *et al*. attempted immunization before pregnancy with a live attenuated vaccine, using tachyzoites of the RH strain with a deletion of the dense granule protein 17 (*GRA17*) gene (Δ*gra17*) that had previously shown to protect mice from lethal infection (32). The deletion entailed a defective parasitophorous vacuole (PV) and decreased intravacuolar tachyzoite proliferation, due to interference with protein transport across the PV membrane. Ip immunization with Δ*gra17* strain elicited the production of Th1-type response cytokines, IL-12, and IFN-γ, as well as of IL-10 in Kunming mice (32). Thereafter, the same authors have tested this vaccine against vertical transmission using both acute and chronic infection models. No maternal clinical signs of infection and abortion were found and the litter sizes of viable neonates in immunized and RH inoculated dams were higher. In both models of infection, pups presented a higher survival rate. Maternal spleen *T. gondii*-induced cytokine production was evaluated at day 18 of pregnancy, 6 days after infection with Pru strain. Higher levels of Th1-type cytokines IFN-γ, IL-12, and IL-2, and of IL-10 were detected. Significantly lower parasite burden was found in the brain of immunized dams. Further, partial protection was observed, concerning brain parasite load in the progeny of these animals (32). Recently, a live-attenuated vaccine of the RH strain was engineered to harbor a deletion of *Gra17* and of novel putative transporter 1 (*NPT1*) gene, encoding a selective arginine transporter (33). The virulence of this strain (RHΔ*gra17*Δ*npt1* **)** was completely attenuated *in vivo*. The brain cyst burden of immunized dams was significantly lower and no abortions were observed compared with non-immunized infected mice. All pups born from immunized infected mice had about 15 times fewer brain cysts than non-immunized infected mice pups (33).

A live-attenuated vaccine using tachyzoites of the Pru strain with a deletion of the calcium-dependent protein kinase 2 (*CDPK2*) gene (Δ*cdpk2*) was also developed by Wang *et al*. (34). CDPKs harbored by *T. gondii* are required for cell invasion and gliding motility and are important virulence factors. Specifically, CDPK2 prevents accumulation of amylopectin to toxic levels in the cell, that would cause the parasite to be morphologically defective and unable to form cysts. Therefore, these parasites were incapable of establishing chronic infection since they were not able to form tissue cysts. Dam brain cyst burden was, in average, 43 times lower than that of non-immunized challenged dams. Splenocytes from immunized dams produced higher levels of IFN-γ, IL-2, IL-12, and IL-10 compared with non-immunized mice when stimulated *in vitro* with soluble tachyzoite antigen. These results indicated that this vaccination approach led to a balanced pro- and counter-inflammatory maternal response, useful to control infection but also to avoid potentially harmful excessive inflammation. Pups from non-immunized infected mice harbored in average 919 ± 339 brain cysts, whereas only 41.4% of the pups from immunized infected mice harbored cysts, averaging 60 ± 33 cyst per brain (34). Recently, Wang *et al*. created a live-attenuated vaccine from the *T. gondii* RH strain with a deletion of the tyrosine kinase-like 1 (*TKL1*) gene (RHΔ*tkl1*) (35). Vaccinated Kunming mice remained without clinical signs of infection and showed significant decrease in brain cyst burden 30 days after delivery. No abortions occurred and litter size was unaltered in immunized mice when infected, while all non-immunized infected mice suffered abortions. A decreased brain cyst load was observed in the pups from immunized infected dams indicating reduced vertical transmission (35).

### Recombinant Protein Vaccines

Recombinant *T. gondii* surface antigen 1 (rSAG1) protein (39) was assessed in two models of congenital infection (**Table 2**). Haumont *et al*. immunized Dunkin-Hartley guinea pigs subcutaneously (sc) with rSAG1 three times at 3-week intervals and intradermally challenged 3 weeks after breeding (25). Vaccination induced protection against maternal-fetal transmission as assessed by the brain parasite load in the live pups. However, the SAG1-specific IgG levels in newborn pups did not correlate with protection, while cellular responses were not evaluated (25). In another study, BALB/c and CBA/J mice (*H2^d^* and *H2^k^* background, respectively) were sc immunized twice with rSAG1 (40). A reduction of 50% of maternal-fetal transmission in BALB/c, but not in CBA/J mice, was observed. Protection found in immunized BALB/c mice correlated with a maternal increase in rSAG1-specific IgG1 and a decrease in rSAG1-specific IgG2a. IFN-γ and IL-10 levels were increased in serum and in supernatants of *T. gondii* lysate antigen (TLA)-stimulated splenocytes obtained from vaccinated animals. In contrast, the immunized CBA/J mice showed no protection and significantly increased serum IL-10 and IL-4 levels. Further, no differences were observed concerning serum IFN-γ or IFN-γ levels in the supernatants of TLA *ex-vivo* stimulated spleen cells from both rSAG1-vaccinated or control CBA/J animals. These observations suggest that the Th1-/Th2-type responses induced by the immunization used were affected by the host genetic background, such as the major histocompatibility complex, leading to the different outcomes after immunization (40).

**Table 2:** Non-live vaccines in vertical *Toxoplasma gondii* infection models.

Strain/animal	Immunogen/delivery	Day of mating post-immunization	ChallengePregnancy day/route/#parasite form/strain	Dam sacrifice	Maternal parameters	Progeny parameters	Highlighted results	References
Dunkin-Hartley/guinea pigs*	Recombinant SAG1/ sc	Day 21	21 Days after mating/id/ 5x10^5^ tachyzoites/ C56 strain	NS	-Specific IgG antibodies at the time of delivery	-Number of litters negative, partially positive, and positive. -Number of stillborn and viable pups and *T. gondii* positive. -Pups brain infectious status 2 days after birth. -Specific neonate IgG antibodies.	-Protection against maternal-fetal transmission (66–86%) -No correlation with specific IgG levels in the newborn pups.	(25)
BALB/c and CBA/J/mice	Recombinant SAG1/ sc	Day 60	Day 12 of pregnancy/oral/ 10 cysts/ Me49 strain	Day 19 of pregnancy	-Maternal SAG1 IgG1 and IgG2a *-Ex-vivo* splenocyte specific cytokine production	-Fetus parasite load	**-**Protection against maternal-fetal transmission in BALB/c mice (50%) -Increased maternal IFN-γ and IL-10 production. Higher maternal rSAG1 specific IgG1 levels in BALB/c mice. - No protection in CBA/J mice associated with a Th2 response.	(40)
BALB/c/mice	SAG1-DNA plasmid/ im	Day 14	Day 12 of pregnancy/oral/ 10 cystes/ Beverley strain	NS	Uterus examination (resorptions) or still births (at delivery)	-Survival and specific IgG in pups 12 weeks after birth -Number of survival pups at day 12 after birth	-No protection against maternal fetal transmission (no differences in pups’s survival and specific IgG levels).	(41)
Swiss OF1/mice	pSAG1mut+pGRA4+pGMCSF DNA plasmids/ im	Day 15	Day 7-10 of pregnancy/oral/ 70 cysts/ 76K strain	NS	NE	-Survival (days 1–30 after birth) - Weight evaluation (days 8, 15, and 30 after birth). - Brain cyst burden (day 30 after birth)	-Higher survival rates but no differences in pups’s body weight. -No protection against parasite vertical transmission (equal brain cyst burden).	(42)
CBA/J/mice	Exosomes derived from DC pulsed with *T. gondii* total antigen sc	Day 14	Day13 of pregnancy/oral/ 25 cysts/ 76K strain	2 months after infection	Brain cyst load	-Survival (days 1–42 after birth) -Weight evaluation every 4 days for 42 days. -Brain cyst burden (6 weeks after birth) -Specific IgG isotypes and IgA response and *ex-vivo* spleen and lymph node specific cytokine production (6 weeks after birth).	- Decreased maternal brain parasite load. - Higher survival rate and pups body weight. - IgG2a, IgG2b, IL-2, IFN-γ, IL-4, and IL-10 responses in the pups. - Decreased pup brain parasite load.	(43)
CBA/J/mice	Di-palmityol phosphatidyl glycerol-loaded nanoparticles (DGNP) containing total *T. gondii* extract/ in	Day 60	Day 11 of pregnancy/oral/ 15 cysts/ 76K strain	Day 17 of pregnancy (exp#1) Day 60 after delivery (exp#2) (6 days post-infection the dams were sacrificed and fetus were collected for parasite load quantification)	*- Ex-vivo* cytokine secretion in placentas (Th1H1: IFN-γ and TNF-α, Th2 (IL4), Treg: TGF-β and IL-10, Th17: IL17A, and anti-inflammatory: IL6) (exo#1). -Brain cyst load evaluated 60 days after delivery (chronic infection) (exp#2).	- Parasites load n fetal tissues by real-time PCR (exp#1). - Evaluation of fetal resorption (exp#1). -Litter size and pups weight (exp#2). -Evaluation of protection in offspring against chronic infection (exp#2). -Average weight of each litter at 7, 14, and 21 days—after birth (exp#2). Ophthalmologic clinical signs (exp#2).	-Decreased maternal brain cyst load. -Reduction of IFN-γ and increase IL-6 and IL-10 placental cytokine levels favoring pregnancy maintenance -Protection against vertical transmission (fetus parasite load) or offspring (brain cyst load) - Higher mean weight and protection against ocular toxoplasmosis in the offspring.	(44)

*Guinea pigs: pregnancy length of 9 weeks. C56 strain: type III; Me49 and 76K strain: type II. sc, subcutaneous; im, intramuscular; in, intranasal; NS, no sacrifice; NE, not evaluated. exp#1: experimental design 1; exp#2: experimental design 2.

### DNA Vaccines

DNA vaccines are among the most promising in *T. gondii* research. These vaccines have numerous advantages such as ease of development, low-cost production, stable storage, and shipping. More than 50 vaccine variants have been experimentally produced and tested and have shown positive results in their protective capacity using exogenous infection models (36, 37). Vaccination with a DNA plasmid encoding SAG1, which previously showed to protect BALB/c mice against infection with the avirulent Beverly type-2 strain, upon intra-muscular immunization with PltPASAG1 plasmid, was tested in a congenital infection model. However, maternal-fetal transmission was not reduced as compared to sham-immunized control mice (41)

This observation led to the conclusion that different immune mechanisms could mediate protection in adult-acquired infection and congenital parasite transmission (41). Another approach was performed by Mevelec *et al*. combining DNA plasmids encoding SAG1, *T. gondii* dense granule antigen 4 (GRA4) and murine granulocyte-macrophage colony-stimulating factor (GM-CSF) (42). The survival rates of pups from immunized infected dams was significantly higher compared to non-immunized infected dams. These results indicate that DNA plasmid multiantigen vaccine works better than a single antigen vaccine (42). Further studies are however necessary to highlight the suitability and efficacy of this type of vaccines in vertical infection.

### Exosome Vaccines

Exosomes are nano-sized vesicles released by most eukaryotic cells (14). These vesicles can contain a wide variety of molecules, such as proteins, lipids, and nucleic acids, able to activate cellular and humoral responses altering the outcome of parasite infections (45).They can transfer mRNA, miRNA, and proteins between cells, representing a communication path between cells, necessary for immune homeostasis (14, 45). Studies showed that immunization with exosomes released from *T. gondii*-pulsed dendritic cells (DCs) induced protection against congenital toxoplasmosis, associated with IFN-γ and IL-10 responses in the pups (43). This vaccine, when administered before pregnancy, provided strong fetus protection against infection. Low cyst burden was observed in both immunized dams and pups delivered from immunized dams (43). This was the first description, so far, studying immune responses in the offspring from vaccinated females.

### Nanoparticle-Based Vaccines

Advances in research have revealed the use of nanoparticles (NP) as antigen delivery systems, thus setting the basis for a new type of vaccines. This antigen delivery system avoids antigen degradation and increases bloodstream life span, internalization, and presentation by antigen-presenting cells, such as DCs (14). Di-palmitoyl phosphatidyl glycerol-loaded nanoparticles (DGNP) loaded with *T. gondii* total antigen extract were shown to deliver parasitic antigens to mucosa after intranasal immunization, inducing a specific Th1/Th17 response *in vivo* (46). Further work has tested the safety and efficiency of DGNP in congenital toxoplasmosis (44). Placental levels of cytokine production were analyzed, which revealed no signs of inflammation exacerbation in immunized mice, even though, there was an increase in IFN-γ concentration. IL-10 and IL-6 levels were significantly raised. Survival of offspring and dams was 100% and mean litter size and pup weight was not diminished in infected immunized mice. The parasite burden on the fetus was 86% reduced from females immunized with DGNT/TE compared to controls (44).

## Conclusion

Most of the research work that addresses vaccination using vertical *T. gondii* infection models assessed the immune response to vaccination, determining the IgG isotypes and cytokines produced in response to parasite antigen stimulation. Typically, production of IFN-γ, IL-2, IL-12, IL-10, and, occasionally, IL-4 was found elevated in response to vaccination in non-pregnant mice until 60–70 days post-vaccination. This might explain why this time-point has been referred in the achievement of pregnant animals, since the balance between these cytokines is essential in successful pregnancy as reported in almost all the studies referred here.

Most reports gather data from acute, chronic, and congenital infection experiments. However, the immune status during pregnancy is altered (7), therefore, the data from non-pregnant mice must be carefully discussed and not directly extrapolated to pregnancy due its specific immune status.

In two studies using live attenuated vaccines, mixed maternal Th1-/Th2-type responses were induced, being discussed as Th1 crucial against *T. gondii* congenital infection and Th2 essential for pregnancy maintenance (32, 34). Currently, a balance between several subsets of T cells must be considered such as Th1, Th2, Th9, Th17, Th22, and follicular Th cells (Tfh) for a successful human pregnancy (47). Indeed, these T cell subsets contribute to the immune response occurring at the maternal-fetal interface, known to be important not only in protecting against infection and controlling inflammatory response, but also contributing to immune homeostasis, implantation, decidualization, maternal immune tolerance and acceptance of the fetus, and parturition (47).

A heterogeneity in the experimental designs was found in the studies reported. The difficulty of performing research work with congenital infection models is very high and may explain the scarcity of research in this area. It would be useful to choose a standard experimental design in the study of vaccines, using congenital models able to get valid and robust results. Murine models are the most chosen because they offer simplified logistics, including the facility to monitor physiologic parameters and the short length of pregnancy. Further, mice allow experiments with higher animal numbers, availability of immunological reagents and genetically modified hosts. Mice were largely validated as an adequate model to study congenital toxoplasmosis, by reproducing many features of human infection (21).

None of the vaccines described so far managed to fully protect against *T. gondii* vertical transmission, even if providing multi-antigenic stimulus. The lack of more effective vaccination approaches may be a consequence of the scarce knowledge on the host protective immune molecular and cellular mechanisms operating at the maternal-fetal interface, specifically at decidua and placenta. Indeed, only one work testing nanoparticles containing *T. gondii* total extracts, analyzed the cytokine profile in placentas from vaccinated dams (44). This study found that a reduction in IFN-γ and an increase in IL-6 and IL-10 production was associated with protection against vertical transmission and ocular toxoplasmosis in the offspring (44). Determining the type of immune response at maternal-fetal interface that can correlate with protection will be useful to refine vaccination approaches, by selecting adjuvants that could adequately polarize T cell responses, thereby leading to protection against congenital infection. On the other hand, it would be also important to understand the immune response developed in the pups born from vaccinated dams and infected during pregnancy. To our knowledge, only one study analyzed the immune response in surviving pups born from vaccinated dams, having found a mixed Th1- and Th2-type response associated with a high survival rate, high weight mean, and low cyst brain burden (43). The development of adaptive fetal immune responses are observed in neonates exposed to an infection environment *in uterus* but not necessarily infected themselves (48). Thus, it will be worthy to get new insights into how T cell responses and related mediators operate in fetuses and in neonates. Novel vaccine design formulations and delivery systems can also be improved, concerning parasite antigen determinants and elicited immune mechanisms involved in protection against *T. gondii* vertical infection (36).

## Author Contributions

MBa, DT, and MBo wrote the initial manuscript. All authors contributed to the article and approved the submitted version.

## Funding

This work was supported by the Applied Molecular Biosciences Unit-UCIBIO, which is financed by national funds from FCT (UIDP/04378/2020 and UIDB/04378/2020). AC was supported by FCT Individual CEEC 2017 Assistant Researcher Grant 352 CEECIND/01514/2017.

## Conflict of Interest

The authors declare that the revision work was conducted in the absence of any commercial or financial relationships that could be construed as a potential conflict of interest.
